# Reconstruction of the subungual region for tumors of the thumb: Outcomes of the reverse, dorsoulnar flap

**DOI:** 10.1016/j.jpra.2025.08.033

**Published:** 2025-09-04

**Authors:** Esin Rothenfluh, Epameinondas Gousopoulos, Esther Vögelin, Radu Olariu, Maurizio Calcagni

**Affiliations:** a Department of Plastic and Hand Surgery, Inselspital, Bern University Hospital, Bern, Switzerland; b Department of Plastic and Hand Surgery, University Hospital Zurich (USZ), Zurich, Switzerland

**Keywords:** Subungual skin tumor, Melanoma, Hand surgery, Thumb homodigital flap

## Abstract

**Background:**

For subungual, malignant skin tumors of the thumb, ultimate treatment has mostly been the amputation, a procedure that comes with a significant functional loss. We propose reconstruction of the defect with the reverse, homodigital, dorsoulnar, pedicled (RHDU) flap, if no osseous infiltration is present.

**Methods:**

We present the cosmetic and functional results in five patients treated for subungual tumors of the thumb between May 2020 and September 2023. Range of motion in the metacarpo—and interphalangeal joints, the widening angle of the first web, Kapandji score, grip and pinch strength, outcome scores including the disability of the arm, shoulder and hand (DASH) and Michigan Hand Score (MHQ) were assessed at final follow up.

**Results:**

Range for follow up was 1–5 years. Function was comparable to the contralateral side. Patient reported outcome showed satisfactory function with an average DASH score of 12.82 and MHQ score of 84.8. The cosmetic result was rated with an average of 90 points on the visual analogue scale for cosmesis. No recurrence of the tumor has been reported in any case.

**Conclusion:**

Reconstruction of the defect with a RHDU flap after excision of subungual tumors and proof of no osseous invasion, is a reliable and a promising alternative to an amputation, resulting in satisfactory functional and cosmetic outcomes.

## Introduction

Subungual malignant skin tumors are rare, with the greatest incidence in the great toe and thumb.^1^^,^^2^ If they affect the thumb, extensive defects down to the distal phalangeal bone after removal of the tumor remain challenging, often resulting in an ultimate ratio management-the amputation. The thumb plays a crucial role in overall hand function, contributing to dexterity and precision. Given this, the functional impact of a distal thumb amputation can be considerable. We propose the reconstruction of the subungual region of the thumb with a reverse, homodigital, dorsoulnar, pedicled flap (RHDU flap), based on the dorsoulnar artery of the thumb. This is a common technique for reconstruction of traumatic dorsal or pulp defects of the thumb and has originally been described by Brunelli et al.^3^^,^^4^ In anatomical studies, the arterial perfusion has been shown to be reliable and is based on the consistent presence of a dorsoulnar branch of the princeps pollicis artery.^4^, ^5^, ^6^ In addition to the anastomosis with the proper digital artery at the middle of the proximal phalanx, there is a vascular arcade at the proximal nailfold, connecting the dorsoulnar with the dorsoradial artery. This arcade is usually sacrificed in cases of subungual melanoma, urging to maintain the proximal anastomosis and to plan precisely in accordance with the defect size and geometry. The donor-side morbidity is generally modest and the flap reliable, if some practical steps are followed. Tension to the pedicle may pose the risk of flap failure in the early postoperative phase. Restricted range of motion in the interphalangeal or metacarpophalangeal joints and constriction of the first web span were reported to potentially result in a less satisfactory outcome.^7^

We present our technical refinements and clinical experience with the RHDU flap in five cases of subungual skin tumors of the thumb. After complete removal of the tumor with clear margins down to bone, we covered the defect of the thumb nail bed with the reverse, dorsoulnar, pedicled flap. The surgical technique, the functional and cosmetic results are discussed.

## Methods

All patients gave institutional general consent for further use of their medical data. All clinical investigations were conducted according to principles expressed in the declaration of Helsinki.

From May 2020 to September 2023 five cases of subungual skin tumors of the thumb in five patients were treated by removal of the nail and surgical tumor excision down to the distal phalanx bone. After subsequent excision biopsies and proof of no marginal residual tumor by histopathological analysis, a reconstruction of the defect with a RHDU flap was performed. Resection in depth was limited by the end phalanx bone, of which none was affected in the presented cases. The tendon of the extensor pollicis longus muscle was maintained. Until reconstruction was performable, all defects were covered by a synthetic skin substitute (EpiGARD, Biovision GmbH).

### Patient characteristics

From May 2022 to September 2023 five patients were treated for subungual skin tumors of the thumb using a sequence of excision biopsy, histopathological analysis, and re-excision or reconstruction with a RHDU flap, depending on the pathology result. Reconstruction was only performed if all margins were tumor-free. The presence of residual tumor cells in the deep margin would have required amputation of the distal phalanx. The demographic characteristics and tumor specifications are summarized in table 1. The patients were treated at two different University centers. They include four men and one woman, the mean age was 63 years (range, 50–73 years) at the time of surgery. Three right and two left thumbs were affected.

**Table 1:** Patient characteristics for each of the five cases.

Case	Follow up time (years)	Age (years)/Gender	Side	Tumor type	Defect size (cm x cm)
1	4.5	64/M	Right	Squamous cell carcinoma	2.5 × 2
2	4.5	50/M	Right	Cutaneous melanoma	3 × 2
3	5	73/F	Right	Lentiginous proliferation	–
4	1	62/M	Left	Cutaneous melanoma	2.5 × 2.7
5	1	67/M	Left	Cutaneous melanoma	3 × 2

Defect size was unavailable for one patient (M = male, F = female).

### Surgical technique

**Figure 1:**
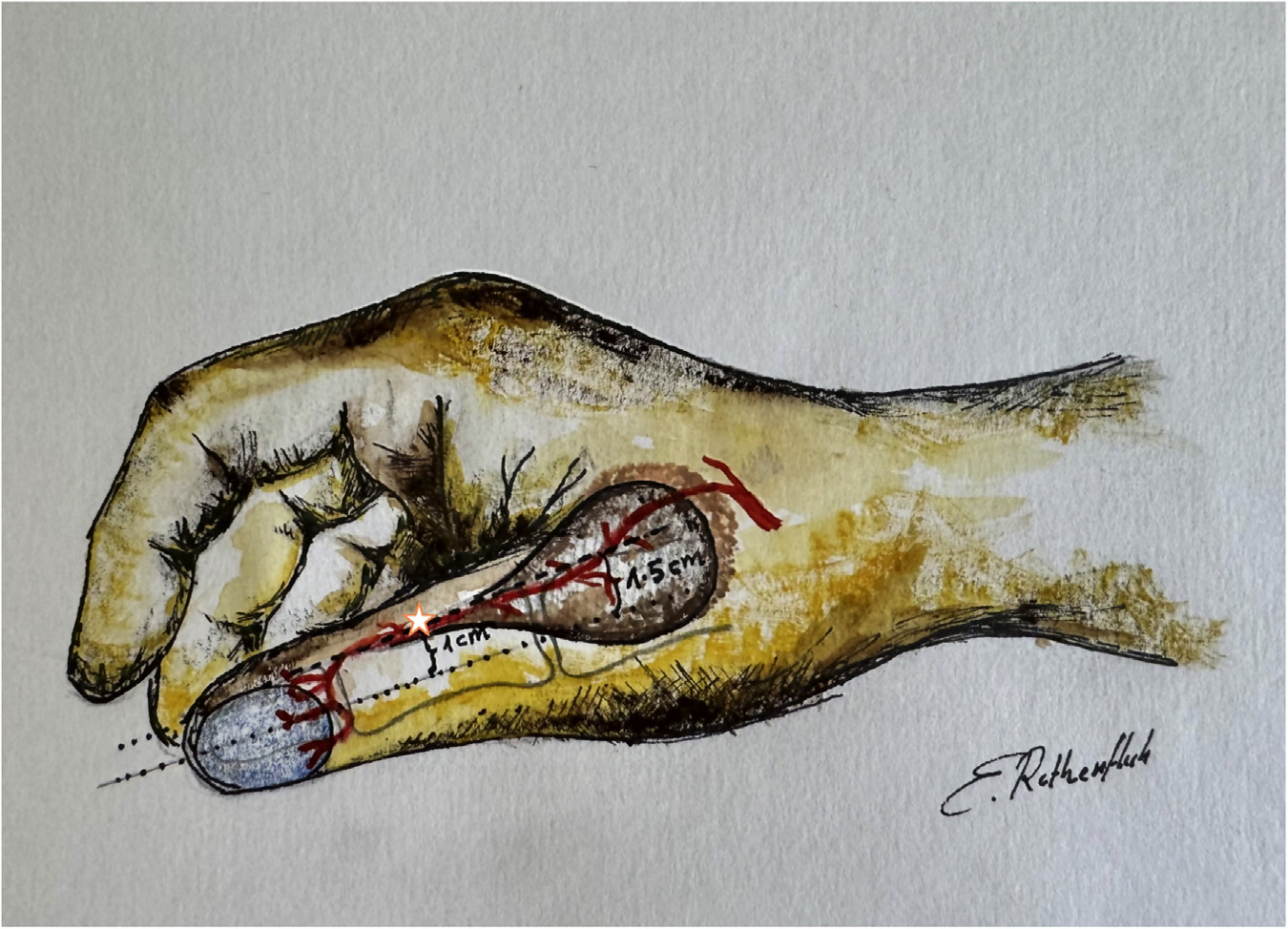
Landmarks for surgical planning of the RHDU flap. The axis of the dorsoulnar artery (dashed line) lies approximately 1 cm from the midline (dotted line) of the thumb and 1.5 cm from the midline towards the base of the thumb. The asterix indicates the pivot point located near the midpoint of the proximal phalanx.

The operations were all carried out under general anaesthesia and with the aid of tourniquet control and loupe magnification. Preoperatively, the dorsoulnar artery was identified with an ultrasound scan and a Doppler probe. However, we did not consider this as a mandatory step, as the dorsoulnar arterial branch is reliably present. The axis of the dorsoulnar artery was marked at the beginning of the procedure with 1–1.5 cm to the midline of the thumb. For the procedure, the flap was harvested on the dorsoulnar aspect of the metacarpophalangeal (MCP) joint, in continuation of the dorsoulnar artery in a proximal to distal fashion (Figure 1). Flap tailoring was carefully planned according to the size of the defect, allowing a tension-free rotation around a pivot point near the middle of the proximal phalanx and ensuring complete coverage of the defect upon inset. We chose to shape the flap like a drop, of which the thinnest part lies over the turning point. This may yield an aesthetically more appealing result. The donor site was closed directly in two cases. A local hatchet-like advancement flap was utilized for closure of the donor site in the three other cases. A full thickness skin graft, taken from the ipsilateral arm, was necessary in addition to the hatchet flap in one of the three cases. While gathering experience and technical finesse with the RHDU flap in this clinical context, but also in other trauma cases, the authors preferred to harvest a flap minimally larger than the actual defect at the distal phalanx. This made a direct closure of the donor site still possible. We used a small skin graft to cover the pedicle strand in the two cases of direct harvest site closure instead of closing the wound in a whole, which would possibly have caused pressure to the pedicle. The thumb was immobilized in a resting position with a palmar splint including the metacarpophalangeal (MCP) and the interphalangeal (IP) joints as well as the wrist for the period of wound healing. After this phase, the MCP joint was mobilized with active and passive flexion—extension exercises, while the IP joint was addressed 2 weeks later. Strengthening exercises were initiated 6 weeks postoperatively.

### Measurement of outcomes

The functional outcome in respect of the range of motion in the MCP and IP joint, the widening angle in the first web space (radial abduction) and Kapandji Score^8^ on a scale from 0 to 10 (0 = no opposition, with the thumb reaching the second metacarpophalangeal joint; 10 = complete opposition, with the thumb reaching the fifth metacarpophalangeal joint) were recorded. The lateral pinch and grip strength were assessed with a finger and hand dynamometer, respectively. All results were compared with the contralateral side. Cosmesis was assessed with the visual analogue scale (VAS) for cosmesis (0 = worst possible scar, 100 = best possible scar). Complications in the early postoperative phase (7 days) and during the whole follow up period were recorded, such as infection, residual nail growth, flap congestion or failure, delay of wound healing, particularly at the donor site, and recurrence of the skin tumor within the observational follow up period. The sensation at the flap site was tested with a two-point discriminator. At the final follow up, a DASH (Disability of the arm, shoulder and hand; range 0–100 with higher scores indicating greater disability) and a MHQ (Michigan Hand Score; range 0–100 with higher scores indicating better outcome for the measured scales) score were taken for the affected hand.

## Results

Follow up time for the five patients were 1–5 years (range 12–60 months, average 38.4 months). For all patients, complete records were available. Patient one and three filled out a short Michigan Score instead of the extended version. One patient had an *in-situ* squamous cell carcinoma (SCC) of the hypertrophic type (Morbus Bowen), three patients had a cutaneous melanoma (one of an acral lentiginous type), and one patient had a lentiginous proliferation with an incontinence of pigmented cells (Table 1)*.* The patient with SCC previously underwent radiotherapy 10 months prior to surgery. Despite this treatment, a recurrence occurred, after which the patient chose surgical management. All skin tumors were located at the thumb`s nail bed. In three cases axillary sentinel lymphnodes were taken at the time of excision of the skin tumor. In one patient, both excised lymphnodes were positive for regional metastasis, prompting a subsequent level I–III lymphadenectomy. In the other two patients, the absence of metastasis was confirmed. No evidence of distant metastasis was found in any of the included patients. Two excisions were necessary until all margins were tumor free in three patients; reconstruction was performed in the third operation. In one patient the sample was tumor free after first excision and reconstruction could be performed in the following procedure. In the patient with no secured melanoma the defect was directly covered with the RHDU flap. Reconstruction was performed within 24 days in average after first excision biopsy (range, 17–31 days). The mean size of the defect was 6 cm^2^ (range, 5–6.75 cm^2^). In one patient no measurement of the defect size could be found retrospectively (Table 2). Consistent data regarding melanoma thickness could not be obtained retrospectively.

**Table 2:** Surgical procedures and complications for each of the five cases.

Case	Excision numbers	Sentinel LN taken	Donor site closure	Complications
1	2	No	hatchet	Infection
2	2	Yes	hatchet + SG	–
3	0	No	hatchet	–
4	1	Yes (LN adenectomy)	direct + SG on pedicle	–
5	2	Yes	direct + SG on pedicle	–

Excision numbers indicate the number of excisions required to achieve clear margins prior to reconstruction.

LN = lymphnode; SG = skin graft.

At the final follow up, average active flexion in the metacarpophalangeal (MCP) joint of the affected thumb was 51° (range 50–60°) and average active extension 6° (range 0–30°). Average active flexion in the interphalangeal joint (IP) was 51° (range 45–60°) and average active extension 16° (range 5–30°). The average ratio of MCP joint flexion on the affected to the non-affected side was 93 %, for MCP joint extension 38 %, for IP joint flexion 78 % and for IP joint extension 100 %. The average widening angle of the first web on the affected side was 56° (range 40–85°) and the average ratio 97 %. The average Kapandji score was 8.6 (range 8–9) on the affected side and the average ratio 93 %. The pinch and grip strength recovered well with nearly equivalent values on both sides in all cases. The average lateral pinch strength was bilaterally 9 kg and the average grip strength 31.4 kg on the affected and 32 kg on the non- affected side. In two patients, sensation recovered at the site of the flap and in one patient sensation was still different with no clear two-point discrimination. There was no sensation at the flap site in the other two patients. Patients rated the cosmetic result with a range of 70–100 (average 90) on the VAS. The detailed measurements at the different time points during follow up are summarized in Table 3. The average DASH score was 12.82 (range 0.8–27.5) and the average MHQ score 84.8 (range 70.8–98.3) (Table 4).

**Table 3:** Functional results. Range of motion in the MCP and IP joints is presented as flexion—neutral—extension.

	MCP	MCP*	IP	IP*	First web	First web*	Kapandji	Kapandji*	Pinch(kg)	Pinch*	Grip(kg)	Grip*
1	50–0–0°	60–0–10°	55–0–20°	80–0–30°	40°	50°	9	10	12	12	33	30
2	40–0–30°	50–0–30°	50–0–10°	55–0–0°	50°	50°	8	8	11	11	49	53
3	55–0–0°	55–0–40°	45–0–30°	60–0–20°	40°	40°	9	10	6	6	21	21
4	50–0–0°	50–0–0°	60–0–10°	60–0–5°	65°	65°	9	9	8	8	20	18
5	60–5–0°	60–5–0°	70–0–15°	45–0–15°	85°	85°	9	8	8	8	36	36

First web refers to radial widening of the first web. The Kapandji score ranges from 0 to 10, with 10 indicating complete opposition. Blank columns represent the right side, those marked with (*) indicate the left side. Values for the tumor side are shaded.

**Table 4:** Patient-reported outcome scores at the final follow-up.

	DASH Score	DASH work	DASH sports/art	MHQ affected hand
1	9.1	n/a	0	70.8
2	0.8	0	0	98.5
3	27.5	n/a	31.3	70.8
4	25.9	25	n/a	91.5
5	0.8	n/a	0	92.4

The DASH work and DASH sports/art scores refer to the two optional modules and are marked with n/a when not applicable. The MHQ score refers to the operated side.

**Figure 2:**
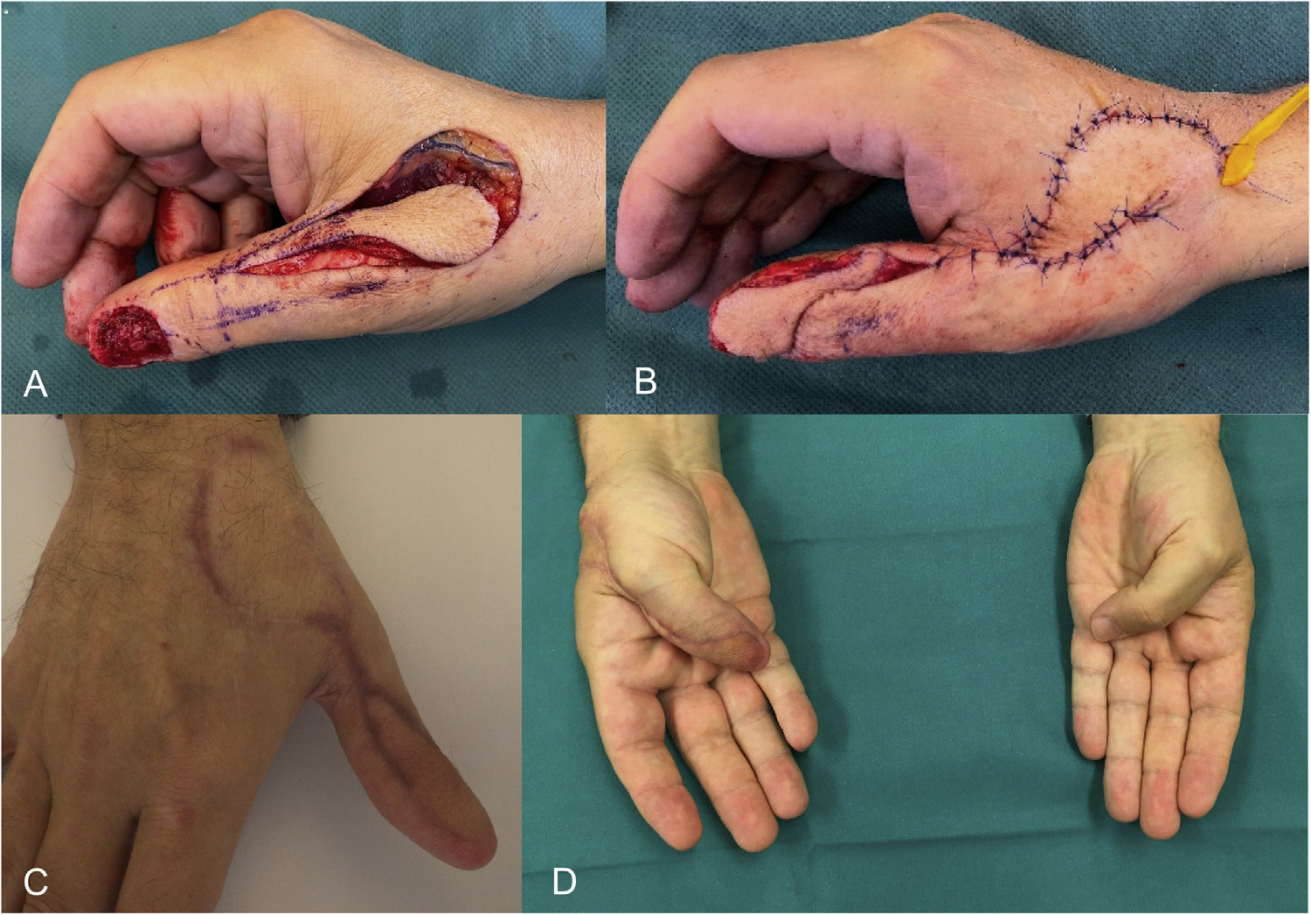
Intraoperative photograph of the flap during preparation for case one (A) and after the inset (B). The donor site was closed with an advancement flap, yielding a satisfactory cosmetic result (C). The degree of postoperative opposition is shown in D.

**Figure 3:**
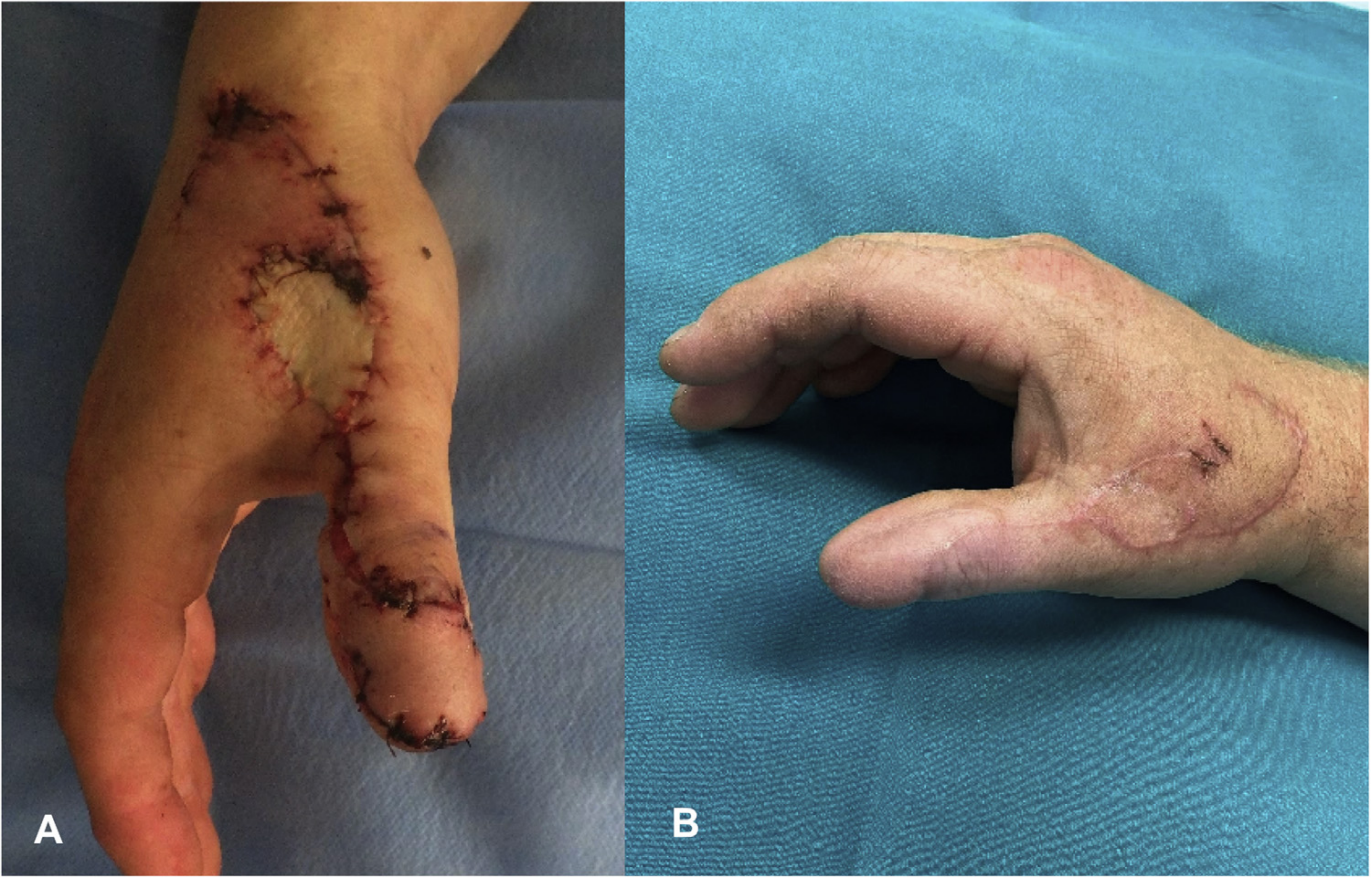
In case two, closure of the donor site was achieved using an advancement flap with a full-thickness skin graft (A), also resulting in a good cosmetic outcome (B).

**Figure 4:**
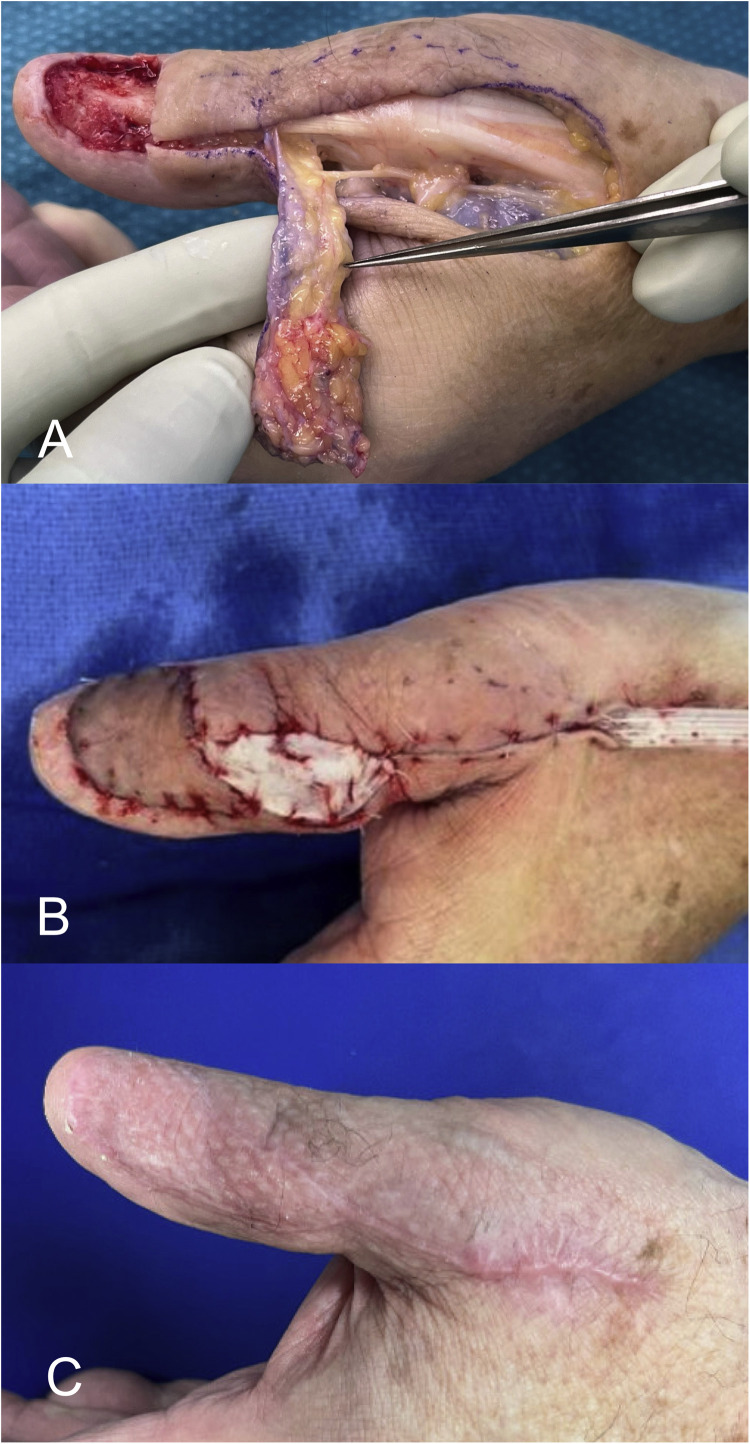
Intraoperative image (A) showing the dorsoulnar artery within the pedicle in case four. The donor site was closed primarily, while the pedicle site was left open and covered with a small full-thickness skin graft (B), resulting in a good cosmetic outcome and wound healing (C).

**Figure 5:**
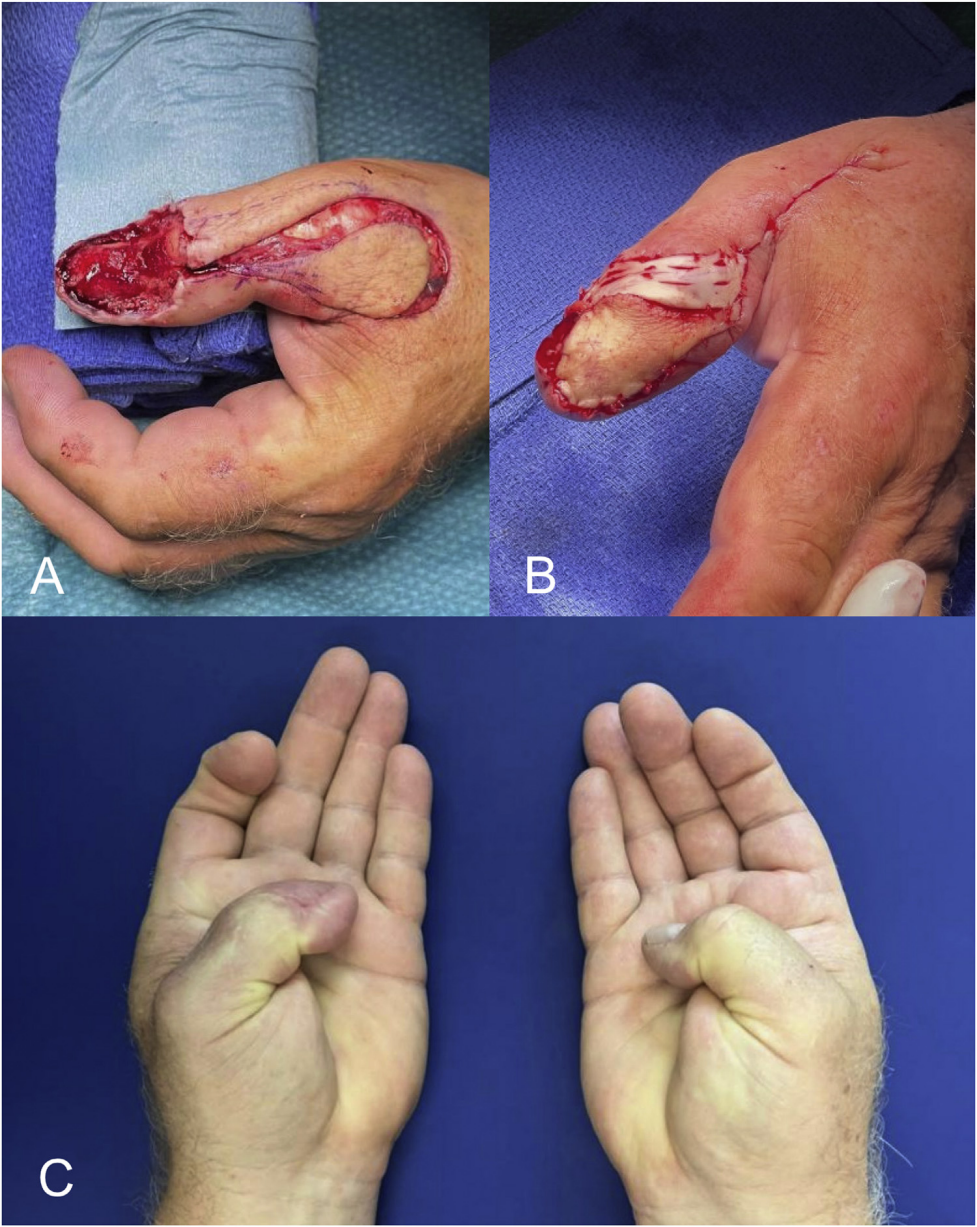
In case five, despite a large defect, adequate coverage was achieved using the RHDU by adhering to the landmarks described in Figure 1 and avoiding extension of the pedicle beyond the pivot point (A). Donor site closure was performed as in case four (B), resulting in satisfactory cosmetic and functional outcomes (C).

In view of complications, one patient had a prolonged wound healing at the harvest site, another patient presented with a residual nail growth 1 year after reconstruction, which caused a local infection. This required a revision surgery. The residual nail could be removed completely, and the patient recovered well. No recurrence of the tumor has been detected in the presented cases to this timepoint. Pre-, intraoperative and postoperative pictures are presented in Figure 2, Figure 3, Figure 4, Figure 5.

## Discussion

Subungual skin tumors are rare, with subungual melanoma representing an uncommon variant, accounting for 1–3 % of all cutaneous melanoma in Caucasians. It appears to be more prevalent among Black, Asian, Native American, and Hispanic populations.^9^^,^^10^ Diagnosis is often challenging due to the tumor`s subtle presentation and the difficulty of obtaining adequate biopsy samples, contributing to its relatively poor prognosis.^1^ Early diagnosis is therefore essential, and biopsy should be considered for nail bed lesions that persist without change for more than 4–6 weeks.^2^ Subungual melanoma most commonly affects the great toe, followed by the thumb, accounting for 75–90 % of cases.^1^^,^^2^ Given the thumb`s critical role in daily funtion, preserving its integrity is important. Previous studies have shown that distal amputations (interphalangeal joints) or local excisions did not compromise overall survival compared to more proximal amputations (metacarpophalangeal joints).^11^ Therefore, radical amputation may not be justified if clear margins can be achieved through local excision.

We propose the RHDU flap for reconstruction and preservation of thumb length following local excision of the nail unit down to bone. It is a reliable flap in terms of vascular anatomy and technical feasibility, though it requires microsurgical experience. We present five cases in this article, in which function of the thumb returned to nearly normal conditions apart from MCP joint extension (38 % compared to contralateral). All patients were satisfied with the appearance and only one had a complicative infection, which could be cleared without consequences. The patient reported outcome scores revealed a good overall function with an MHQ above 70 in all cases, of which three even had a score of 95 and more.

We optimized the technique by closing the donor site primarily or with an advancement flap, leaving the pedicle`s rotation point open and covering it with a small full thickness skin graft to avoid tension that could impair flap perfusion or cause congestion. The flap was not innervated, but the dorsoulnar nerve branch was preserved to prevent neuroma formation. Interestingly, in at least two patients, sensation recovered—possibly due to axonal ingrowth from the recipient site, supported by the flap’s small size and limited thickness, which have been identified as relevant factors in previous studies.^12^ Three of the presented cases involved subungual melanoma and one involved SCC. Subungual SCC is similarly rare and often diagnosed late, as it can mimic benign conditions. Although bone involvement is reported more frequently in SCC, this was not observed in our case.^13^ In the fifth case no malignant skin tumor was found. Histology revealed a lentiginous proliferation with pigment incontinence and the patient was managed similarly to those with subungual melanoma.

In the literature, clinical experience with the RHDU flap is mainly based on trauma cases. As an alternative to the RHDU flap, Chen et al.^14^ performed reconstruction of the thumb tip in context of degloving injuries with a modified first dorsal metacarpal artery flap, which was raised from the index finger and supplied by the vascular chain between the first dorsal metacarpal artery and PDA. This reconstruction method however may compromise function, sensation and appearance of the index finger. The biodegradable temporizing matrix (BTM) has recently emerged as a promising, cost-effective alternative for managing fingertip injuries involving nail bed damage. However, published clinical report remain limited and focus primarily on small sterile matrix defects.^15^ Various reconstructive options have been described after thumb amputation, including the composite osteocuteanous groin flap and the chimeric radial collateral artery perforator flap.^16^^,^^17^ While these techniques allow thumb reconstruction, they involve long operative times and require advanced microsurgical skills. Additionally, patients must undergo a complex treatment course with two major surgeries. In cases of osseous tumor infiltration, such approaches remain valid.

The main limitation of this study is the small number of patients. However, subungual melanoma is rare, making it difficult to draw authoritative conclusions about the optimal treatment algorithm. Nevertheless, when surgical clearance is achieved, the RHDU flap appears to be a favorable solution for reconstruction of the thumb nail bed, allowing preservation of thumb length and avoiding distal thumb amputation.

## Conclusion

Reconstruction of the nail bed defect with a RHDU flap after complete excision of skin tumors with histopathological proof of no osseous invasion, proves to be a favorable solution and a promising alternative to an amputation, resulting in satisfactory functional and cosmetic outcomes.

## Declaration of competing interest

The authors declare no potential conflicts of interest with respect to the research, authorship and publication of this article.
